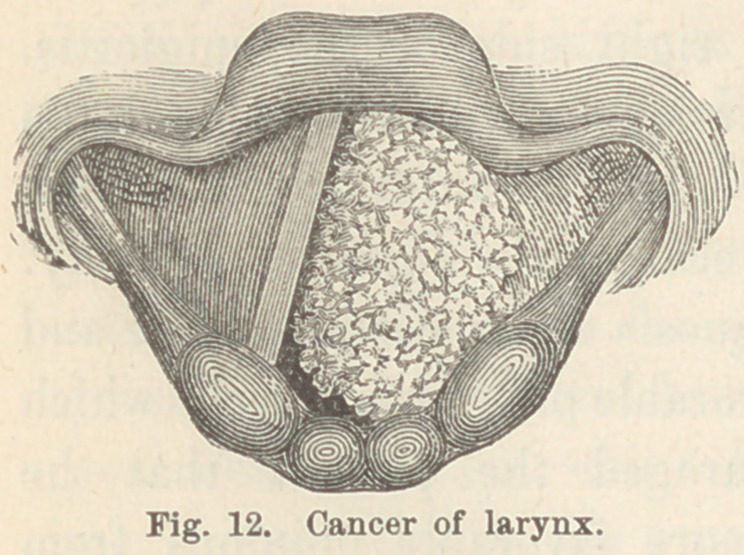# Laryngeal Tumors

**Published:** 1881-07

**Authors:** E. Fletcher Ingals

**Affiliations:** Lecturer on Diseases of the Chest and Physical Diagnosis, and on Laryngology, Rush Medical College; Professor of Diseases of the Throat and Chest, Woman’s Medical College, Chicago


ARTICLE II.

LARYNGEAL TUMORS. Report to the Illinois State Medical Society by E. FLETCHER INGALS, A.M., M.D., Lecturer on Diseases of the Chest and Physical Diagnosis, and on Laryngology, Rush Medical College; Professor of Diseases of the Throat and Chest, Woman's Medical College, Chicago, May 18, 1881.

Less than a quarter of a century has elapsed since the discovery of the laryngoscope removed the diagnosis and treatment of diseases of the throat from the field of empiricism, and placed it among the most exact specialties of our art.

No more striking illustration of the benefits that have accrued from a knowledge of laryngoscopy can be found than that which is contained in the history of laryngeal growths.

For twenty-five hundred years preceding the year 1857, not more than seventy cases had been recorded, where either ante-mortem chance or post-mortem investigation had, by the discovery of a morbid growth, revealed the true nature of many obstinate and fatal cases of laryngeal disease. Since the experiments of Czermak and Türck many of these growths have been discovered; hundreds of patients have been relieved, and many have been rescued from death by the removal of tumors which would otherwise have caused strangulation. Nearly every variety of morbid growth which may affect the human body has been discovered in the larynx, but the great majority of intra-laryngeal tumors are of a papillary character. Probably ninety-seven or ninety-eight per cent. of all these growths are benign in nature.

Of all laryngeal tumors the papillomata constitute about seventy-five per cent.; the fibromata about twelve per cent., and fibro-cellular growths about five per cent. Of the remaining, the greater part are cystic, and after these, in the order of their frequency, come the sarcomata and lipomata, together with rare instances of mucoid, vascular or adenoid growths, and finally cancers. Of primary cancer it has been my misfortune to see three cases.

Morbid growths in the larynx are found most frequently in males; they may occur at any age, but between the ages of twenty and forty, they are more common than at either extreme of life. Of my own patients, the youngest was six years old and the oldest seventy.

Excepting the malignant growths, these tumors are generally the result of chronic catarrhal inflammation of the larynx, of a mild character. They are occasionally caused by syphilis, and not infrequently by phthisis. In some instances measles, croup, diphtheria, whooping-cough, or the inhalation of irritating substances seem to have acted as exciting causes.

The symptoms caused by these growths, depend mainly upon their location and size, and are much the same regardless of the exact nature of the tumor. The patient usually gives a history of having had a severe cold, contracted several months beforehand, from which he has never fully recovered. There has generally been some hoarseness at first, which has at times been better and at times worse, until finally it has become persistent; but in some cases the aphonia continues paroxysmal for a long time. The hoarseness may progress to complete aphonia, and if the tumor is large, considerable dyspnœa may be experienced. The affection of the voice is often most marked with small tumors, especially if they are attached to the vocal cord. Often these patients complain of a tickling sensation in the throat, and when the tumor is pedunculated they frequently experience sensations like those produced by a foreign body in the larynx.

The growth seldom causes much pain, but frequently it gives rise to slight discomfort, especially on swallowing. If the tumor is of considerable size the difficulty in deglutition may be very marked. Even with small growths, speaking is often tiresome, and with the larger it may be nearly impossible, either from the impediment to the free vibration of the cords or from lack of force in the expiratory current of air.

Respiration is often stridulous when the tumor is large. Cough is usually present, but it varies greatly in character and frequency. It may be harsh and dry, or easy and loose, and it is sometimes croupy. In some cases there is scarcely any cough, while in others this may be the most distressing symptom. With small neoplasms the expectoration is usually slight, but with the larger growths, whether benign or malignant, it is frequently excessive. In these latter cases collections in the larynx of tenacious mucus greatly add to the suffering and danger of the patient. This mucus may cause great difficulty in respiration and doubtless in cases which are not properly treated it is often the immediate cause of death.

*Diagnosis.*—By auscultation over the larynx or trachea a moist râle or sort of valvular murmur may sometimes be detected, but even if all the ordinary symptoms and signs of a tumor are discovered, an accurate diagnosis cannot be made without the laryngoscope.

By the aid of a small mirror, placed in the throat, and a good light reflected upon it, we can usually at once determine the nature of the difficulty; though in some cases the intractability of the patient or the peculiar location of the tumor may necessitate repeated examinations.

*Prognosis.*—The prognosis in cases of benign laryngeal tumors depends upon their size and location. If a tumor is small and located above the vocal cords it may give the patient no particular inconvenience, but if situated on the cord it causes more or less aphonia.

Tumors as large as a pea usually cause aphonia, even though located above the vocal cords.

The tendency with most of these growths is to gradually increase in size, though some of them, after attaining a certain size, may remain stationary for years.

When a tumor has once caused hoarseness there can be no reasonable hope for the disappearance of this symptom until the growth has been removed.

Large growths, by which I refer to tumors varying from the size of a pea to that of a filbert, often jeopardize the patient's life. They may do this by exciting and rendering permanent a harrassing cough which may gradually exhaust the patient; or, in consequence of the small size of the glottis they may so interfere with respiration as to cause sudden death by choking, or more gradual dissolution through the deleterious effects of continuous imperfect aëration of the blood; or their pernicious effects may be mainly due to the difficulty which they cause in deglutition.

Malignant tumors in the larynx, so far as past experience goes, are fatal. Treatment may prolong life for a few days or months, but a time will soon come when deglutition or respiration will become impossible, and then tracheotomy or even extirpation of the larynx can add only a brief span to the patient's existence.

*Treatment.*—There are two plans of treating benign laryngeal tumors. First, that calculated to relieve the local hyperæmia, and, second, that for the destruction or removal of the growth.

A few laryngologists discourage all operative procedure so long as the growth does not materially interfere with the individual's means of obtaining a livelihood, or directly endanger life; and these believe that the sole treatment in many cases should be that adapted to chronic laryngitis ; for example, the topical application of strong mineral astringents or mild caustics.

While this treatment is undoubtedly adapted to some cases, it should be accorded a secondary place. It is often useful as an adjunct to operative measures, but it will seldom effect a cure. I have seen tumors considerably diminish in consequence of the persistent use of such remedies, but they have soon attained their original size when the treatment was suspended, or even during its continuance. In some cases I have no doubt that such treatment stimulates the tumor to more rapid growth.

Notwithstanding the difficulties and dangers of operative procedures, I am fully in accord with those laryngologists who believe that, as a rule, benign growths in the larynx should be removed by operative measures. This should be accomplished through the natural passages when possible; and when this is impracticable, if the growth endangers life, it must be removed by tracheotomy, by thyrotomy, by supra-thyroid laryngotomy, or by division of the thyro-hyoid membrane, or by infra-thyroid laryngotomy.

However, extra-laryngeal methods should not be adopted even when endo-laryngeal methods cannot be carried out, unless the patient's life is endangered by the presence of the tumor.

Most of the papillary growths found in the larynx are not larger than a pea, but occasionally they attain the size of a walnut. They are generally multiple. These tumors are usually attached to the vocal cords, to the ventricular bands, or to the inter-arytenoid fold. They may be pedunculated, but they are more apt to be sessile.

Most papillomatous tumors of the larynx are of a light pinkish color, and have a granular surface or are laminated like condylomatous growths. They are soft and friable, so that they may be easily crushed or pulled off with forceps. Voltolini has shown that they may occasionally be detached by frequent up-and-down movements of a sponge passed into the larynx.

Tumors of this kind are not likely to recover after they have been thoroughly removed, excepting in phthisical patients.

As has been stated, papillomata constitute about three fourths of all laryngeal tumors, and their immediate cause is a chronic hyperæmia of the mucous membrane from which they spring.

To illustrate the earlier stages of their growth, I will cite three cases of chronic laryngitis, in which the circumscribed swelling of the mucous membrane indicates the beginning of what may ultimately become a well defined tumor.

OUT-GROWTHS.

CASE I. *Out-growths from the vocal cord.* J. K., æt. 50, laborer. This patient came to me about a year ago, complaining of hoarseness and severe cough. The lungs and heart were found to be healthy. Laryngoscopic examination revealed general diffuse inflammation of the mucous membrane of the larynx.

Fig. 1.—Outgrowth from right vocal cord.

After a few astringent applications the patient passed from my observation. About three months ago he returned with much the same symptoms as at first. During the interim he had been sometimes nearly well, and sometimes quite hoarse. At this time I found the larynx greatly congested, and the vocal cords red and thickened. Upon the free edge of the right cord, about its middle, was an outgrowth of a conical form, the apex of which projected about four millimeters into the rima-glottidis (Fig. 1.) The base of this swelling extended six or eight millimeters along the free edge of the cord.

This case illustrates the origin of many laryngeal growths. At first there occurs chronic catarrhal inflammation of the part, which is followed by an excessive proliferation of cells in the sub-epithelial connective tissue, which finally results in a morbid growth supplied with new blood-vessels, and covered with an attenuated epithelium. The tumor thus formed may be single, but more often is made up of numerous papillæ, each of which may give off secondary or tertiary offsets, which give to the whole mass a strawberry-like or cauliflower appearance. The former occurs when the epithelial covering encloses the whole mass, the latter when it is insufficient to fill the spaces between the papillæ.

In cases of this kind the proper treatment consists of frequent applications to the larynx, by means of a brush, of some strong astringent, such for example, as: Argenti nitras, gr. xl—lx *ad* aquam ℥i; zinci sulpas, gr. xxx—lx *ad* aquam ℥i; zinci chloridum, gr. xx—xxx *ad* ℥i, or liq. ferri per chlorid., min. xv—xxx *ad* aquam ℥i. At the same time sedative inhalations or internal remedies may be needed to relieve cough. In addition to these, benefit will sometimes be derived from stimulating inhalations which may be employed by the patient at his home.

In the case of this patient I have obtained the best results from applications of sulphate of zinc, gr. xxx *ad* ℥i, and the internal use of bromide of potassium with small doses of belladonna. Inhalations could not be employed. His visits have been irregular and the treatment has been correspondingly unsatisfactory; but the congestion has been much relieved, the prominence on the vocal cord has been considerably reduced in size, and the voice is correspondingly improved.

Case II. *Incipient laryngeal tumor.* J. S., æt. 43, printer. When this patient first consulted me he had been hoarse for six months. He had been troubled most of the time with cough, but his general health had been good until a few weeks before I saw him.

Inspection of the larynx revealed swelling of the mucous membrane just beneath the right vocal cord at its posterior extremity (Fig. 2.) The prominence extended along the side of the larynx

Fig. 3.—Incipient laryngeal tumor.

for nearly a centimeter, and stood out beyond the vocal cord about four millimeters. The mucous membrane covering the laryngeal side of the inter-arytenoid fold presented a notched and yellowish gray appearance, strongly suggestive of ulceration below the part which was visible.

Treatment.—Astringent applications were made with the laryngeal brush and by the insufflator, and tonics were given internally.

The patient was soon obliged to discontinue his attendance at my office on account of his work, so that the effects of treatment could not be demonstrated.

CASE III. *Subglottic œdema, resulting from chronic laryngitis, and having the appearance of incipient tumors.* W. T., æt. 65, farmer. The patient stated that he first had trouble with his throat twenty-nine years ago, as the result of measles. At that time hoarseness lasted two years. About eight years later hoarseness again returned and continued troublesome for several years. He has had repeated attacks of eczema for twenty years, and for the past eighteen months has suffered from it constantly. When the patient consulted me he was very hoarse and complained of some dyspnœa on exertion, and of constant though slight sore throat.

Upon laryngoscopic examination I found the right ventricular band so much swollen that it nearly hid the right vocal cord. The mucous membrane below the anterior extremity of the left cord, and that below the posterior extremity of the right cord were swollen so as to present the appearance of morbid growths, and to considerably interfere with respiration. The condition of the throat was apparently due to the same cause as the inflammation of the skin.

PAPILLARY GROWTHS.

CASE IV. *Papilloma of larynx.* P. S., æt. 28, butcher. This patient stated that about three years before consulting me he had several attacks of sore throat which left him with hoarseness which had been constantly present for two years. I could obtain no history of either phthisis or syphilis. The lungs and heart were normal.

Fig. 3. Papilloma on right vocal cord.

Laryngoscopic examination revealed a tumor about the size of a large pea, on the right vocal cord near its anterior extremity. The tumor was of a light red color; it had a slightly granular surface and it was attached to the free edge of the cord by a large pedicle.

At the first sitting I removed, with common laryngeal forceps, about half of the growth, and at the second sitting, three days later, I removed all that remained and cauterized the base with the solid nitrate of silver. To prevent inflammation I directed the application to the neck of hot applications, which were to be continued as long as there was any soreness of the larynx. At the patient's next visit, three days later, the vocal cords were still congested. Soreness of the larynx had lasted but a few hours after the operation. At this time I applied to the cords a stimulating solution of chloride of zinc gr. v. *ad.* aquam ℥i, and ordered bitter tonics to correct some gastric disturbance.

The patient was not seen again for a month; at that time the voice was perfect in ordinary conversation, but not reliable for singing. The vocal cords were still of a pink color. I then applied a solution of sulphate of zinc, gr. xx. *ad.* aquam ℥i, and ordered the daily use of a spray from a four-grain solution of the same remedy. About six weeks later the patient came again to my office, when I found the vocal cords of a natural color and the cure complete.

CASE V. *Tumor of the left ventricular band.* J. S. M., æt. 28, physician. When this gentleman consulted me he had been troubled with hoarseness for several months. An examination revealed chronic inflammation of the pharynx with a highly congested state of the larynx.

The left vocal cord was nearly hidden from view by the swollen ventricular band, and on the posterior half of the same band I discovered a sessile growth of a light color, ovoid in form, about six millimeters long, and projecting about three millimeters from the surface.

The patient's throat was too irritable for immediate operation, and as he was obliged to leave the city the same evening, nothing was done. I have not seen him since, but he informs me by letter, that his throat seems in the same condition, and that he has done nothing for it.

CASE VI. *Papilloma of larynx.* Mrs. B. S., æt. about 25. Ten years ago this patient was nearly suffocated in consequence of laryngeal tumors. These were removed by Prof. Wm. Bruns, of Tübingen, and according to the patient's statement, the larynx was thoroughly cauterized with nitrate of silver and the galvano-caustic. Afterward the patient had no more trouble until a few months since, when she began to complain of a sensation as of a bolus in the throat.

Upon examination of the larynx, I discovered a small, semitransparent growth at the anterior end of the right vocal cord. This was about four millimeters in length, by two or three in breadth, and it protruded from the upper surface of the cord about two millimeters. A papillary growth was also found on the inner side of the upper part of the left arytenoid cartilage.

The smaller growth I destroyed by the solid nitrate of silver. The larger I removed with forceps, and then thoroughly cauterized its base with solid nitrate of silver.

The removal of the tumor, in this case as in most others, caused little pain; but the caustic was very painful. Hot fomentations were directed to be kept constantly applied to the neck, for from twenty-four to thirty-six hours, or until all soreness had disappeared. In this patient the papillæ at the base of the tongue were much enlarged, and they doubtless had something to do with the sensation of a foreign substance in the throat. This case illustrates the occasional tendency of papillomatous growths to repullulation.

CASE VII. *Papilloma of larynx.* A. J., æt. 39, machinist. This patient stated that he had been more or less hoarse for three months before coming to see me. He had never had syphilis and had no knowledge of a hereditary predisposition to consumption. He occasionally had slight pain on swallowing and he sometimes suffered from choking spells. I found the patient's digestive organs in good condition; he was well nourished and had no fever. His lungs and heart were healthy. Upon examining the larynx I found the epiglottis congested, but not swollen; the arytenoids slightly swollen, and the vocal cords slightly congested. A large papillary growth about seven millimeters in breadth by five millimeters in altitude, and one and one-half centimeters in length, occupied the laryngeal side of the inter-arytenoid fold and hung between the cords.

Fig. 4. Papilloma on inter-arytenoid fold.

This tumor though as large as an ordinary raisin, was completely hidden during phonation, and owing to the pendant position of the epiglottis it could seldom be seen at other times. Owing to difficulty in getting this patient to inspire easily and thus expose the growth, I thought it advisable not to attempt its removal until by topical applications the congestion of the parts had been partially relieved and the larynx had become tolerant of instruments.

Only a few applications were made to the larynx when the patient discontinued his visits to my office, but he has lately returned, and I have on two occasions introduced forceps into the larynx, but I have not yet been able to reach the growth. Although this tumor hangs very low in the larynx, I expect to seize it as soon as manipulation renders the patient's throat a little more tolerant.

CASE VIII. *Papilloma on under surface of epiglottis.* E. C., æt. 24; brick manufacturer. Two years before calling on me this patient had pneumonia in the upper lobe of the right lung, and he had been subject to frequent attacks of intermittent fever. Three months before consulting me he had an attack of pleurisy from which he recovered, but ever since he had been troubled with sore throat and hoarseness. The latter had been most annoying in the morning. He had been very much troubled at night with cough though there has been only a small amount of mucus expectorated. When I first saw him he had slight pain at intervals on deglutition, and, in consequence of this pain and broken rest, had lost six or seven pounds during the last twelve weeks. I found that he had never suffered from specific disease, and none of his relatives so far as known, had died of consumption. The apex of the right lung showed slight evidence of consolidation, but there was no dullness on percussion.

Upon examining the larynx, I found an omega-like epiglottis, swollen to twice its normal thickness, with a small spherical papillary growth about six millimeters in diameter on its under surface near the vocal cords. The ary-epiglottic folds were thickened and slightly pyriform in shape, and the whole inner surface of the lower half of the epiglottis, of the anterior end of the ventricular bands, and of portions of the true cords, had a granular appearance with here and there erosion of the mucous membrane. From the respiratory sounds and the appearance of the larynx, I made the diagnosis of laryngeal phthisis.

Treatment: The larynx was penciled with a solution of morphia, gr. iv, carbolic acid, gr. xxv, and tannic acid, gr. xxx, in equal parts of glycerine and water sufficient to make one ounce. This had the effect of relieving pain, of giving immunity from cough at night and thus securing rest, and of somewhat reducing the swelling of the larynx. The papilloma was removed with forceps and the larynx was touched three or four times with solid nitrate of silver.

At the end of the first week the patient was directed to inhale the compound tincture of benzoin, morning and evening (ʒi, to aq. Oi, at 150° F.) He was subsequently given cod liver oil. The local applications to the larynx were continued as at first with the substitution now and then of a solution of chloride of zinc, gr. xv to ℥i of water. I find the following notes in my case book after two weeks of treatment: pulse 104, temperature 100° F.; appetite fair and cough moderate; patient has gained three pounds in flesh.

At this time he returned to his home in Indiana, with the advice that he continue the inhalations and cod liver oil, and as soon as practicable make a change of climate. I have recently learned from my friend Dr. Cassidy, of South Bend, Indiana, that this patient shortly after returning to his home went to Austin,

Texas, where he died quite suddenly about four months later. The immediate cause of death could not be ascertained.

CASE IX. *Tumor on right vocal cord.* R. D., æt. six years. I learned that this little patient had been apparently perfectly healthy since he was two months old, but three months before he was brought to me his voice began to fail, and at the time I saw him there was complete aphonia, the patient being unable to talk except in a whisper. After considerable difficulty, arising from the intractibility of the patient, and a pendent epiglottis, I obtained a view of the larynx, which revealed congestion of the cords and a small sessile tumor near the middle of the right vocal cord on its free edge. (Fig. 5.) There appeared to be some growth below the cord but it was impossible to make a satisfactory examination. As there was no dyspnœa an operation did not seem advisable.

Fig. 5. Tumor on right vocal cord.

In cases like this, operative procedures are not advisable unless, from increase in the size of the growth, dyspnœa should become marked, then tracheotomy should be performed. Then, if the tumor cannot be removed through the mouth, thyrotomy is likely to become necessary. Internal remedies may be of service in lessening the congestion of the mucous membranes and in retarding the growth of the tumor, and astringent or slightly stimulating inhalations should be employed for the same purpose.

FIBROMATA.

Fibrous tumors of the larynx are usually of small size and are generally located on the vocal cords; sometimes, however, they attain the size of a cherry. They may be attached to other parts of the larynx.

These growths are usually rounded in outline, single, and pedunculated, but they may be nodular as though made up of several tumors bound together by an investing membrane, and they are sometimes sessile.

They are usually of a grayish white or red color. They grow slowly, and when once removed have no tendency to return. One of these tumors, which I treated, was developed under my own observation, in a young lady affected with slight catarrhal laryngitis. The history of the case is as follows:

CASE X. *Fibrous tumor of left vocal cord.* Miss D. H., æt. 22. This patient came to me complaining of hoarseness, which, as determined by a laryngoscopic examination, was caused by simple catarrhal inflammation of the larynx. Her general health was good, and after being treated a few days, her voice was so much improved that she discontinued her visits and did not return for several months. This time I found a small fibrous growth attached to the free edge of the vocal cord near its anterior extremity. (Fig. 6.)

Fig. 6. Fibroma of left vocal cord.

Astringent applications were made to the larynx from time to time for several weeks, with the effect of relieving the inflammation of the parts surrounding the growth, and at times apparently reducing the size of the tumor itself; but notwithstanding the apparent improvement, the tumor grew until it reached the size of a small pea. I then attempted its removal by means of Mackenzie's tube forceps. The throat was very sensitive at first, and it was not until after numerous sittings that I succeeded in grasping the tumor, and then, owing to its firmness, only a part of its mucous covering was removed. I immediately seized the growth again and crushed it as thoroughly as possible between the blades of the forceps. The patient was then sent home with instructions to guard against taking cold, and to return in four days. The operation caused but little pain.

On her next visit, there was still some swelling of the vocal cord, but the tumor had disappeared. There was still hoarseness, but the voice was improved. The patient experienced some pain in swallowing, the evening after the operation, but this disappeared in a few hours.

When I saw her again, two months after the operation, all swelling of the cord had disappeared, and she informed me that the voice had been perfect for several weeks.

CASE XI. *Tumor of the larynx apparently fibroid.* Mr. J. M., æt. 63. Upon consulting me this patient stated that his voice had been weak for six or seven years; he had been troubled more or less with hoarseness for four or five years and for the past three years he had been constantly hoarse. For three months he had been troubled with dyspnœa, and for the last three weeks all the symptoms had been aggravated. The difficulty in speaking seemed mainly due to deficient force of the expiratory current of air. On laryngoscopic examination a large pyriform growth was found immediately beneath the vocal cords; against which it was pressed in phonation. (Fig. 7.)

Fig. 7. Sub-glottic tumor of larynx.

The tumor was of a pale-pink color, was about eight millimeters in diameter, and was attached to the anterior surface of the larynx by a large pedicle. An operation was recommended but the patient declined to have the growth interfered with, stating that he had lived with it for several years and thought he could do so several years more.

FIBRO-CELLULAR TUMORS.

Fibro-cellular tumors are sometimes classed as soft fibromata. They are rare. They grow slowly, but may attain a large size. When removed they have no tendency to recur. They consist of delicate fibro-cellular structure, the insterstices of which are filled with fluid or semi-solid granular matter containing nucleated cells. One small growth of this character has fallen under my observation, and I have treated another which seemed to me of the same nature, though I could not be certain in the diagnosis. In both cases, the notes of which are given below, I removed the tumor.

CASE XII. *Small fibro-cellular growth on anterior extremity of right vocal cord.* Mrs. L. S., æt. 20. This patient came to me complaining of slight hoarseness and inability to use the voice for more than a few minutes at a time. She also noticed, occasionally, a slight dull pain in the throat. The difficulty dated from a slight cold fifteen months previously.

Upon examination of the larynx I found the epiglottis hanging so far back as to render it difficult to expose the anterior extrem-

Fig. 8. Tumor on right vocal cord.

ities of the vocal cords; but during the phonation of a high-pitched *a*, I could see a small growth on the free edge of the right vocal cord, about four millimeters from its anterior end. This growth was about the size of a hemp seed, sessile and made up of three small nodules. (Fig. 8.)

The growth was removed with Mackenzie's tube forceps. Three days later no trace of it remained excepting slight congestion of the vocal cords; and in ten or twelve days the cure was complete, the voice having regained its normal condition. Over two years have now elapsed without any return of the laryngeal trouble.

CASE XIII. *Small sessile fibro-cellular tumor on left vocal cord.* Mr. E. B., aged 30. When I was first consulted, this patient had been slightly affected with nasal catarrh for about three years, and had been troubled with hoarseness for several months. I found a sessile tumor on the lower surface and free edge of the left vocal cord, about two millimeters from its anterior extremity. (Fig. 9.) The growth was about five millimeters in length, and projected about two millimeters beyond the free margin of the cord. The mucous membrane of the post-nasal space and of the pharynx was considerably relaxed.

Fig. 9. Tumor on left vocal cord.

This patient's throat was so sensitive that several sittings were necessary before instruments could be tolerated. Astringents were applied at each sitting, and finally the tumor was removed with Mackenzie's common laryngeal forceps. Ten days later no trace of the growth could be seen, and in about six weeks the voice was perfectly natural.

### CYSTIC TUMORS.

Cysts are seldom found in the larynx. Those which have been observed have generally sprung from the epiglottis or from one of the ventricles. They may attain a large size.

Contrary to what would be expected from our knowledge of other retention cysts, these tumors are not likely to return if they are thoroughly laid open, their contents emptied out, and the cavity cauterized with solid nitrate of silver.

CASE XIV. *Cystic tumor.* Mr. B., æt. 20. The only growth of this kind with which I have met, occurred in the larynx of a young farmer who could not remain in the city long enough for operative procedures. The patient had been hoarse for several months but was not troubled with cough or dyspnœa. A laryngoscopic examination revealed a small cystic growth, about eight millimeters in diameter at the posterior extremity of the right ventricular band.

The patient's throat was too sensitive for immediate operation, and, as he was obliged to return to the country on the following day, no effort was made to open the cyst.

### CANCER OF THE LARYNX.

In the early stages of cancer of the larynx the diagnosis is often doubtful, as at this time the symptoms due to the laryngeal tumor are not usually different from those caused by benign growths, and often the essential cachexia does not present itself. Pain, dyspnœa and dysphagia are often present, but these symptoms vary greatly in different cases according to the seat, size, and condition of the growth. Fauvel states that at first the pain is confined to the larynx and that it does not radiate to the ears until ulceration takes place.

In one of my cases the acute, burning pain commencing in the larynx, radiating to the upper part of the pharynx, and later, to the right ear and right side of the head, together with the presence of a large nodular tumor involving the ventricular band, ary-epiglottic fold and epiglottis, left no doubt as to the nature of the case. But, in the other cases, nothing either in the symptoms and general condition of the patient, or in the physical appearance of the growth enabled me to make an exact diagnosis. In these cases at first the physical appearance of the growth was so much like that of an ordinary papilloma that only a microscopical examination by an experienced pathologist could determine its true nature. Even then the fact that many tumors of the larynx which have had a malignant appearance to the microscopist, have had a benign history and course, induced me to hope that these might possibly be of a benign character; however, in both instances the subsequent history has justified our worst misgivings.

For cases of laryngeal cancer there can be but one prognosis, as thus far all that have been reported, with three exceptions one of which was probably non-malignant at first, have died within a few months after the disease became developed sufficiently to cause the patient to seek advice. Mackenzie states that the usual duration of epithelioma of the larynx is about eighteen months, and of encephaloid three years.

The forms of treatment which must be considered in cases of laryngeal cancer are: removal through the natural passages and thorough cauterization of the base of the growth; tracheotomy; thyrotomy; and extirpation of the larynx.

In cases where there is a well defined tumor, of doubtful character the first of these methods seems preferable; but when the growth is not well defined and when it springs from the sub-mucous tissues, or where it involves a considerable portion of the larynx, this method cannot effect its complete removal, therefore, one of the other methods must be tried if anything is done.

Tracheotomy will usually add several months to the patient's life, and has prolonged it in some cases for two years.

Thyrotomy.—The results of thyrotomy with removal of the growth have been very unsatisfactory. In some cases where this operation has been attempted, it could not be completed; in others the patients have died in a few days, and in nearly all of the remaining cases the growth has speedily returned.

Extirpation of the larynx may be practiced in suitable cases, but it is an operation attended with great danger, in which, as stated by Dr. P. Kock, "The skill of the surgeon is, in some cases, shown by the patient not dying under his knife." I find records of twenty cases in which this operation has been performed. Of these, eight died in from two to fourteen days; one in six weeks, and in eight the disease returned and proved fatal in a few months. Of the three remaining cases one died of phthisis a year and a half after the operation; and in the other two there has been no return of the affection.

All things considered, when endo-laryngeal treatment cannot be successful, tracheotomy seems to hold out the greatest encouragement to both surgeon and patient; but even this in most cases should not be strongly urged by the surgeon as at best it can only add a few months to a miserable existence. One of the arguments used in favor of the removal of cancers which can be easily reached is, that when they return they may affect some vital organ and thus terminate the patient's life without prolonged suffering. If this argument was applied to cases of cancer of the larynx even tracheotomy would never be advised.

CASE XV. *Cancer of larynx.* S. P., æt. 69. This patient came to me complaining of hoarseness and great dyspnœa. The former had lasted eighteen months, the latter had been present six weeks.

Upon examination a large growth was found on the right side of the larynx apparently springing from the ventricular band, and extending along its whole length. About three-fourths of the glottis was obstructed by this tumor. (Fig. 10.)

Fig. 10. Cancer of larynx.

Owing to an omega-like epiglottis and to difficulty in getting the tongue sufficiently out of the mouth, I had great difficulty in obtaining a good view of the growth, and subsequently I found even greater difficulty in introducing forceps for its removal.

At one of the first sittings a portion, the size of a large pea, was removed and submitted to Prof. Danforth for microscopic examination. He pronounced it a semi-malignant growth likely to return. Several sittings were necessary before the tumor was entirely removed. At the end of six weeks my notes state that although the growth had been removed the patient complained of great weakness; for which he was given tonics. Three weeks later I was called to see him at his home. He was greatly prostrated, and, on examination of the larynx, I found a swelling about the size of a filbert, which had the appearance of an abscess of the right ventricular band. This was incised freely with laryngeal lancet two or three times with the effect of materially reducing its size though no pus escaped. Subsequently fungoid granulations sprang up and grew so rapidly that in two or three days they nearly stopped the glottis. These were removed every second or third day for about two weeks. At that time the submucous tissues became involved more extensively and the obstruction of the larynx could not longer be relieved by forceps.

After consultation with Drs. H. A. Johnson and E. Ingals, it was decided that no considerable relief could be afforded without tracheotomy.

The case was fully explained to the patient but he hesitated about the operation, and death terminated his sufferings about thirty-six hours later.*

CASE XVI. *Cancer of larynx.* P. N., æt. 59, laborer. Four months before consulting me, this patient began to have slight burning pains in the throat, on deglutition. Shortly afterward these pains frequently occurred at other times, and finally they became nearly continuous, with frequent exacerbations. The pain began in the lower part of the larynx from which it would dart to the upper part of the pharynx, and finally to the right ear and the right side of the head. Hoarsness had been present for nearly four months. At this time the patient's appetite was fair and the general health seemed as good as usual. He did not suffer from dyspnœa when quiet excepting while lying down.

An examination of the larynx revealed a large nodular growth about two centimeters in diameter which involved the right ventricular band, the right arytenoid cartillage and ary-epiglottic fold and about one-fourth of the right side of the epiglottis. (Fig. 11.) The tumor hid the posterior four fifths of the glottis from view, but the anterior extremities of the vocal cords appeared healthy. The diagnosis of cancer was made and an unfavorable prognosis given ; which so discouraged the patient that he sought more favorable opinions from other physicians. Two months later I was called to see this patient at his home. The growth had then ulcerated and great destruction of

Fig. 11. Cancer of larynx.

* This case was at first supposed to be non-malignant, and was reported as such to the Chicago Medical Society.

the upper parts of the larynx had occurred. The patient was extremely weak, was suffering greatly, and was unable to eat. He had failed rapidly in the last two weeks. Anodynes were given and the friends informed that the end was near.

I have been unable to learn the subsequent history of this case.

CASE XVII. *Cancer of larynx.* R. S., æt. 53, farmer. Tumor on left ventricular band. When this patient first consulted me last September I found that he had been hoarse for eighteen months, and had suffered considerably from cough, which occasionally caused pain. There was no dyspnœa and no difficulty in deglutition.

Upon examining the larynx I found a large whitish growth with a granular surface, which extended the whole length of the left ventricular band from which it seemed to spring. The tumor projected from the surface nearly a centimeter. It hid the left vocal cord completely and covered about one-third of the glottis. An attempt to remove the growth with the ordinary laryngeal forceps failed on account of its firmness, but two or three pieces as large as peas were removed and a microscopic examination of these by Prof. Danforth, led to the conclusion that the growth was of a semi-malignant character. The patient was obliged to return to the country before any further operative procedures could be instituted. He was then given iodide of potassium freely for several months.

This patient returned to the city last week. Since I saw him in September, 1880, the tumor had grown so as nearly to obstruct the view of the glottis. Only the right vocal cord and a small part of the rima glottidis, about two millimeters in width, could be seen. (Fig. 12.) Dyspnœa had become quite constant, and the aphonia was much increased.

Fig. 12. Cancer of larynx.

By means of laryngeal knives and forceps, I removed at the first sitting, about two-thirds of that portion of the growth which was visible.

Two days later the patient returned, and I then discovered that the growth extended downward some distance below the vocal cords. With the knives and forceps I now removed a large part of the obstructing mass so as to leave a free opening for respiration.

Prof. I. N. Danforth has examined portions of the tumor removed at the first sitting. He states that the growth has passed the semi-malignant period and that it is now a true cancer.

Prof. Bridge has examined some of the pieces removed at the second sitting and corroborates this serious diagnosis.

P. S.—This growth was removed as completely as possible, with laryngeal knives and forceps, and its base was thoroughly cauterized with solid nitrate of silver.

When he returned to his home the respiration was easy, the glottis being about three-fourths its normal size. His physician, Dr. H. Reineking, was requested to open the trachea as soon as dyspnœa again becomes urgent.

P. S.—Just as this was going to press I received a letter from Dr. R., stating that a few days after the patient's return, he found the sub-glottic portion of the growth somewhat enlarged since the operation. Twelve days later the enlargement was so great as to considerably obstruct respiration, and on the twelfth of June, about three weeks after the operation, he had been called in the night to see the patient, who was having great difficulty in respiration. Perceiving that tracheotomy would be necessary, he sent for another physician to assist him in the operation, but the messenger had hardly left the house when the patient ceased breathing.

The doctor promptly opened the trachea with no assistance save that of a few laborers, breathing was restored, and now the patient bids fair to make a good recovery from the tracheotomy.

## Figures and Tables

**Fig. 1. f1:**
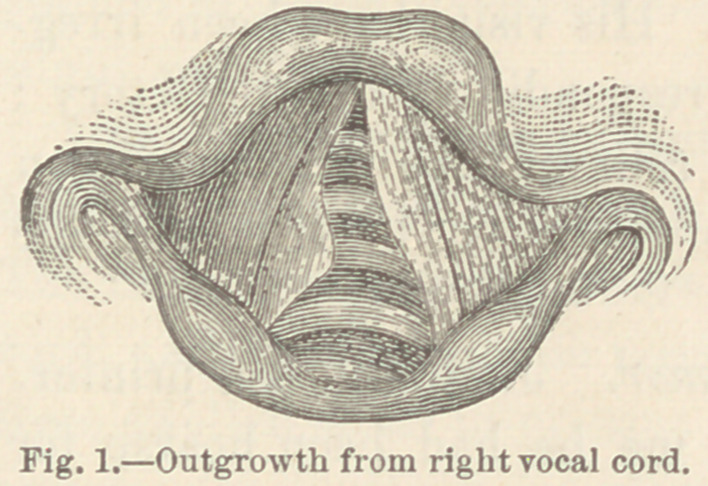


**Fig. 3. f2:**
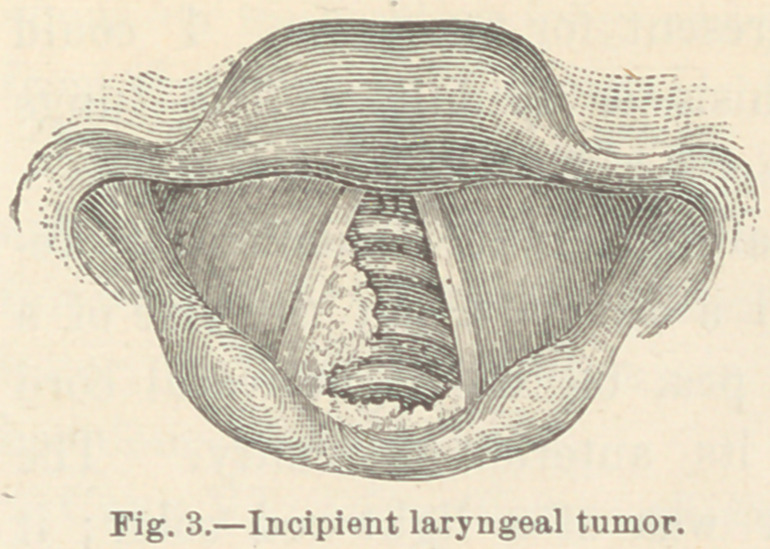


**Fig. 3. f3:**
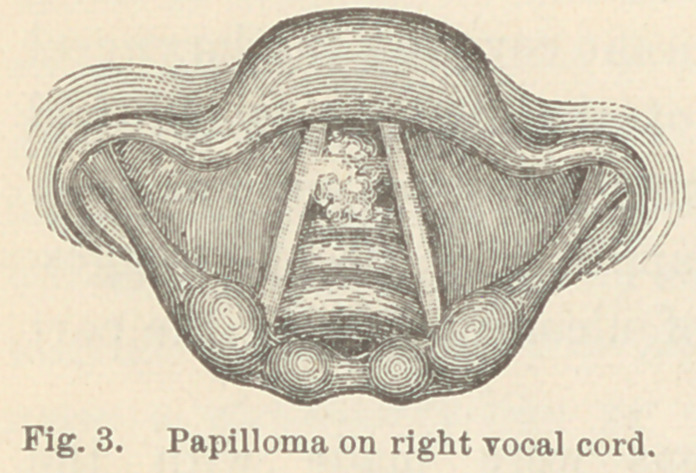


**Fig. 4. f4:**
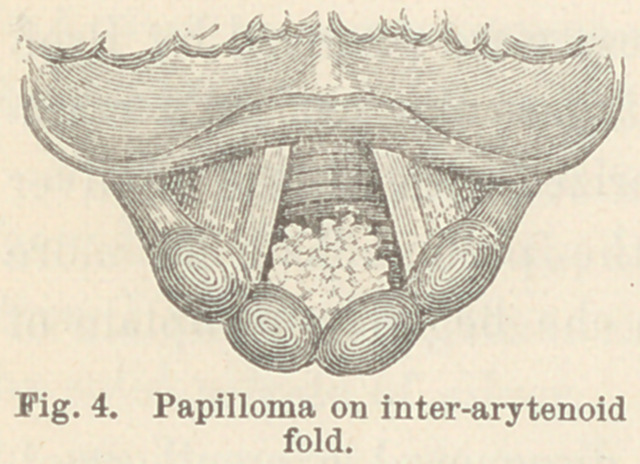


**Fig. 5. f5:**
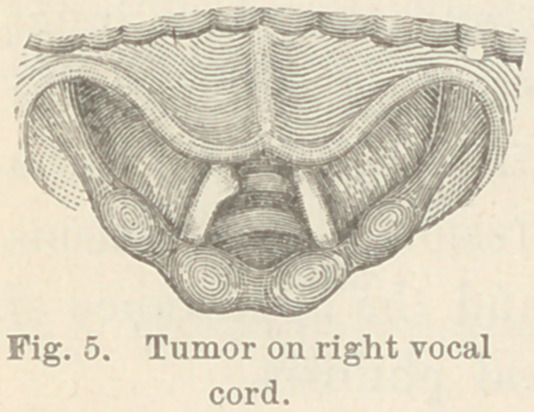


**Fig. 6. f6:**
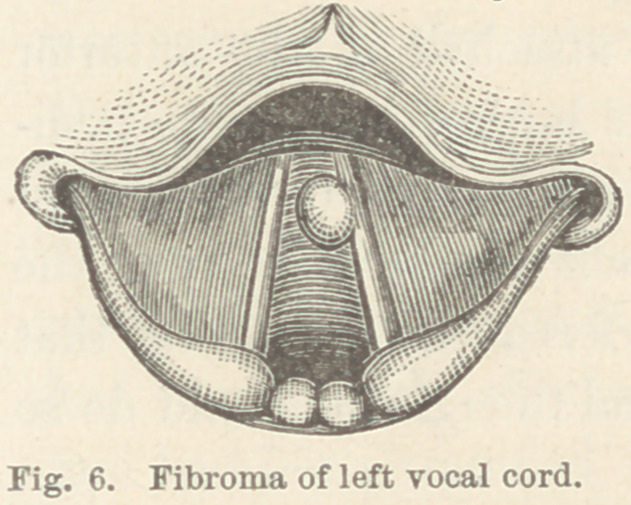


**Fig. 7. f7:**
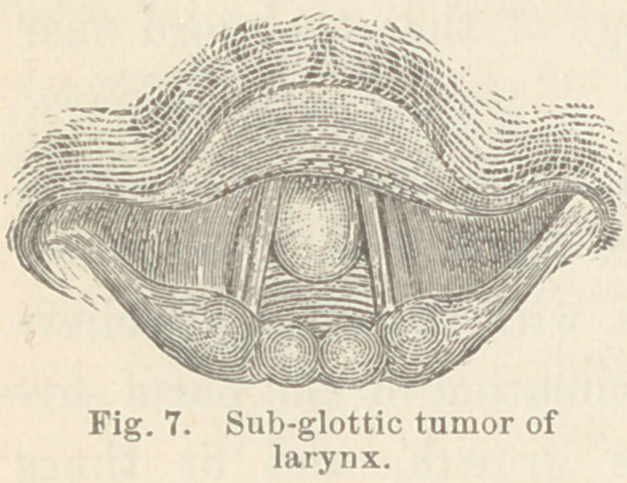


**Fig. 8. f8:**
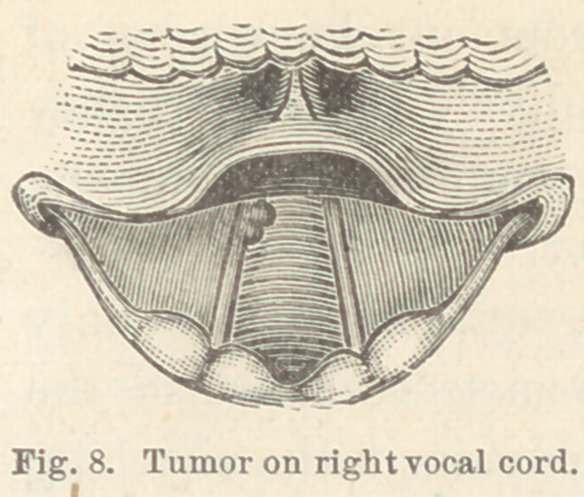


**Fig. 9. f9:**
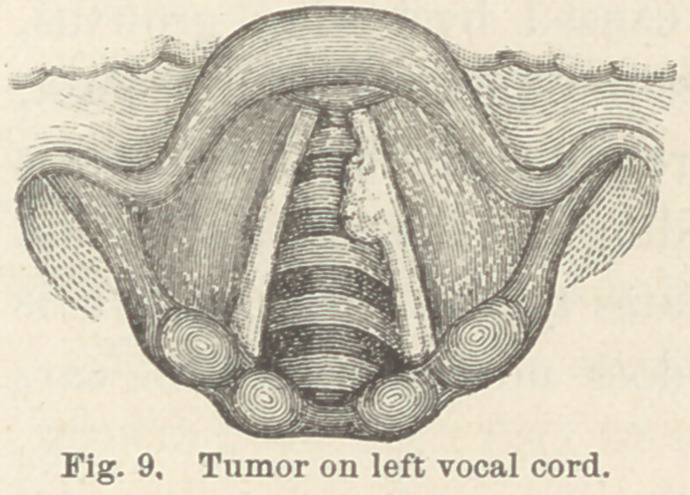


**Fig. 10. f10:**
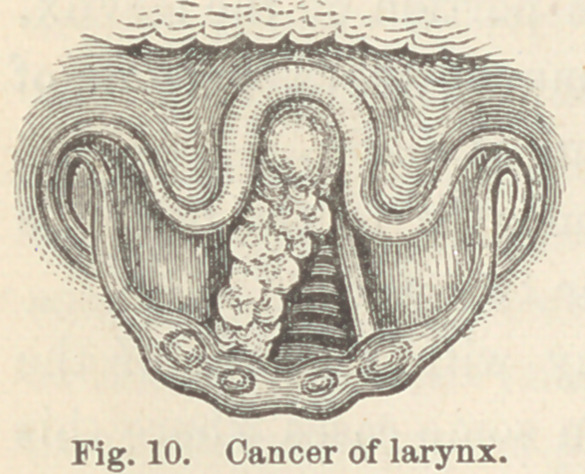


**Fig. 11. f11:**
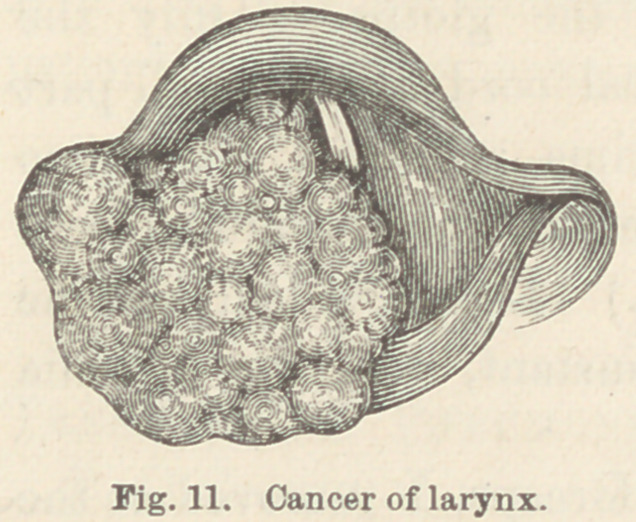


**Fig. 12. f12:**